# Biopolymeric Prodrug Systems as Potential Antineoplastic Therapy

**DOI:** 10.3390/pharmaceutics14091773

**Published:** 2022-08-25

**Authors:** Adriana Aurelia Chis, Anca Maria Arseniu, Claudiu Morgovan, Carmen Maximiliana Dobrea, Adina Frum, Anca Maria Juncan, Anca Butuca, Steliana Ghibu, Felicia Gabriela Gligor, Luca Liviu Rus

**Affiliations:** 1 Preclinical Department, Faculty of Medicine, “Lucian Blaga” University of Sibiu, 550169 Sibiu, Romania; 2 Department of Pharmacology, Physiology and Pathophysiology, Faculty of Pharmacy, “Iuliu Haţieganu” University of Medicine and Pharmacy, 400349 Cluj-Napoca, Romania

**Keywords:** biopolymer, prodrug systems, PDEPT, antineoplastic therapy, chitosan, hyaluronic acid, dextran, pullulan, silk fibroin, heparin, *Auricularia auricula* polysaccharides

## Abstract

Nowadays, cancer represents a major public health issue, a substantial economic issue, and a burden for society. Limited by numerous disadvantages, conventional chemotherapy is being replaced by new strategies targeting tumor cells. In this context, therapies based on biopolymer prodrug systems represent a promising alternative for improving the pharmacokinetic and pharmacologic properties of drugs and reducing their toxicity. The polymer-directed enzyme prodrug therapy is based on tumor cell targeting and release of the drug using polymer–drug and polymer–enzyme conjugates. In addition, current trends are oriented towards natural sources. They are biocompatible, biodegradable, and represent a valuable and renewable source. Therefore, numerous antitumor molecules have been conjugated with natural polymers. The present manuscript highlights the latest research focused on polymer–drug conjugates containing natural polymers such as chitosan, hyaluronic acid, dextran, pullulan, silk fibroin, heparin, and polysaccharides from *Auricularia auricula*.

## 1. Introduction

Cancer remains one of the leading causes of death worldwide, with approximately 19.3 million new cancer cases and 10 million deaths in 2020 [1]. Cancer therapy includes conventional treatments such as surgery, radiotherapy, and chemotherapy and advanced and innovative treatments, such as stem cell therapy, ablation therapy, gene therapy, targeted drug therapy, etc. [2]. Conventional chemotherapy represents the most commonly used treatment in most types of cancers [3], although there are some limitations related to it, such as: narrow therapeutic indices, high-dose requirements, severe adverse effects, low oral bioavailability, limited water solubility, emergence of multidrug resistance and lack of specific targeting [4,5].

Various strategies focusing on improving the selectivity of cytotoxic compounds by targeting the delivery and activation of prodrugs inside the tumor tissue have been addressed [6,7]. Such strategies are polymer-directed enzyme prodrug therapy (PDEPT) that uses polymer–drug and polymer–enzyme conjugates [8], antibody-directed prodrug therapy (ADEPT) that allows the activation of prodrugs by specific enzymes delivered to cancer sites by the use of monoclonal antibodies [9,10], gene-directed enzyme prodrug therapy (GDEPT) in which the cytotoxic drug is activated inside the tumor cells by using an exogenous enzyme encoding gene, and virus-directed enzyme prodrug therapy (VDEPT) in which viral vectors are employed [11,12,13,14].

This selective activation of drugs is achieved by exploiting essential characteristics of neoplastic pathology, such as specific enzymes, low extracellular pH, or hypoxia. The strategic theory of using a specific trigger to activate a prodrug was developed in the late 1980s by Bagshawe et al. on ADEPT and by Huber et al. on VDEPT and GDEPT [15,16]. Later, Satchi et al. have shown a new mechanism of activation of the drug that depends on certain conditions or stimuli, known as PDEPT, in which the polymer–enzyme conjugate boosts the release of the drug molecule from a polymeric conjugate [17]. In addition to exploiting these strategies to release the active drug from prodrugs, a novel polymeric enzyme-loaded nanoreactor has been reported to selectively release the active drug into the tumor [18].

A characteristic of the PDEPT strategy is represented by the release of the active drug that is not dependent on the rate of internalization of the conjugate or the intracellular level of the activating enzyme [8]. The same strategy was later applied to liposomal formulations, by developing liposomal therapy with polymeric enzymes (polymer enzyme liposome therapy—PELT), where the drug is released from liposomes in the interstitial tumor [19].

**Figure 1:**
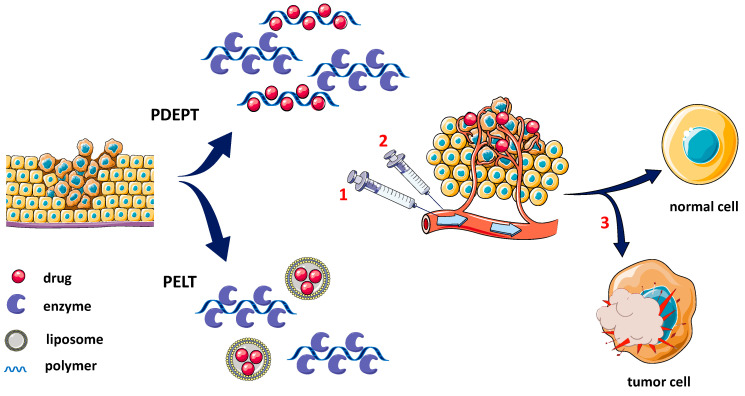
Stages in PDEPT and PELT strategies: 1—administration of polymer–drug/lipid–drug conjugate; 2—administration of polymer–enzyme conjugate; 3—intratumoral drug release. Adapted from [22], Elsevier, 2017.

The polymeric conjugates for drug delivery consist of three elements: solubilizer units, a targeting fragment, and a therapeutic drug, all of them being covalently incorporated into the structure of the polymer. This model was first proposed by Helmut Ringsdorf in 1975 [20,21]. Thus, PDEPT and PELT offer a new approach to antitumor therapy in two main categories, namely the use of a mixture of polymer/liposome–drug and polymer–enzyme conjugates to release a cytotoxic drug rapidly and selectively at the site of the tumor. The major advantage of these strategies is the outsourcing of the release of the active drug, with the accumulation and selective local intratumoral delivery [22]. Both PDEPT and PELT strategies are carried out in two steps: initially the polymeric prodrug is administered to facilitate tumor targeting before the second step of administration of the active polymer–enzyme conjugate (Figure 1).

The process described in Figure 1 uses the enhanced permeability and retention (EPR) effect that targets the prodrug system, as well as the conjugated polymeric enzyme [23]. Nanoparticles of liposomal drugs and polymer–drug conjugates (PDCs) accumulate in the tumor cells through a mechanism of extravasation (with increased permeability and amplified retention by the EPR effect) and the active molecule is released into the cytoplasm [22].

**Figure 2:**
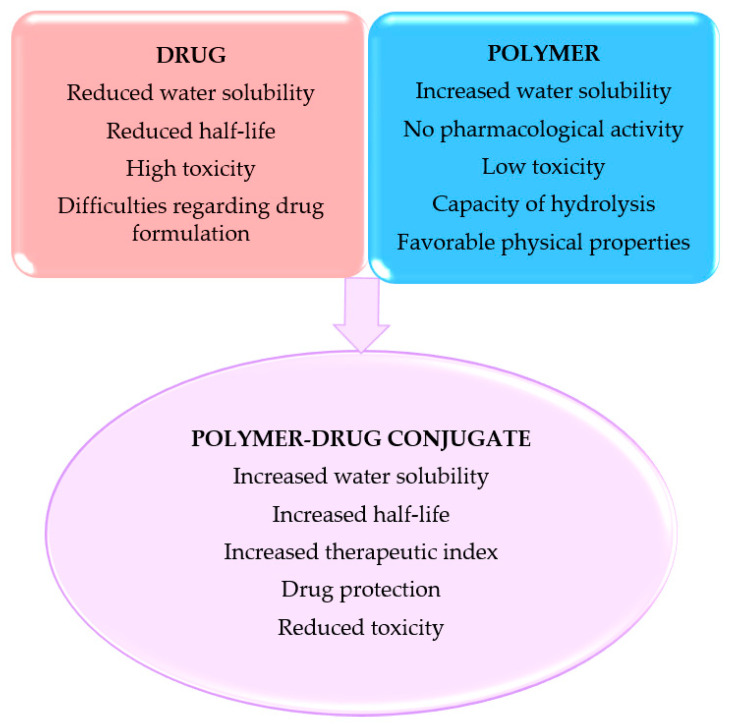
Improved properties of polymer–drug conjugate [8,22].

PDCs offer some major therapeutic advantages, such as [8,22] (Figure 2):

- low toxicity and immunogenicity;
- relatively short residence time in the plasma circulation permitting the following enzyme–polymer conjugate administration, unaccompanied by the drug release in circulation;
- increased half-life and establish dosing intervals;
- improved pharmacokinetics and bioavailability;
- increased drug solubility;
- controlled drug release over long time periods, in recurrent and adjustable doses.

The PDEPT strategy has brought important benefits in therapy because it has compiled expertise on polymer–enzyme conjugation (to enhance stability and decrease immune reactions) and polymer–drug conjugation in drug therapy and especially in antineoplastic therapy, with compounds in the clinical research phase [24]. The development of this polymer therapy represents a new step for targeted therapy in a selected site of action [25]. Malignancy heterogeneity attributed to variability of tumor dimensions, vascularity, and the action of immune system components may influence the biodistribution and accumulation of macromolecular drugs, exploring the characteristic microenvironment of the tumor to achieve drug release [26,27].

One of the first studies conducted on the PELT therapeutic strategies described polymer-phospholipase C compounds used for the release of anthracycline from the liposomal formulation. The phospholipase C was conjugated with 2-hydroxypropyl methacrylate (HPMA) [19,28]. This enzyme promoted the degradation of the lipid membrane of the liposomes, followed by an increase in the release of the active pharmaceutical ingredient from these formulations and to the maintenance of the enzymatic activity after the conjugation with the HPMA copolymer [29].

Therapeutic polymers can be regarded among the first nanomaterials, which can be defined as aggregates or nanoscale complexes, containing minimum two components, one of which is an active agent. The polymers used in these therapies are of high quality and respect the most important desideratum: (i) to be non-toxic; (ii) water-soluble; (iii) biologically inert; (iv) completely biodegradable; (v) to give a certain form of physical protection to the drug; and (vi) to be linked with the drug through a hydrolysable bond [30].

**Figure 3:**
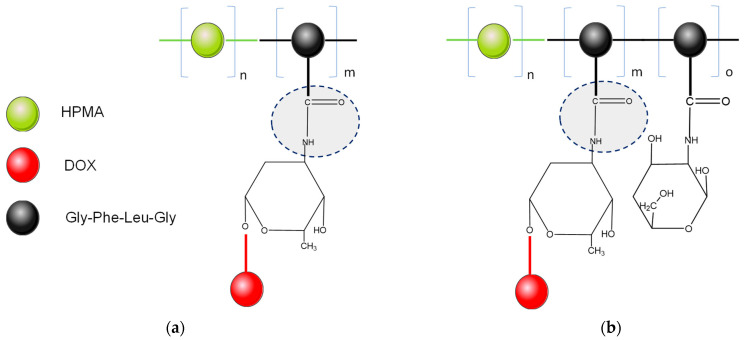
Examples of doxorubicin and HPMA conjugates: (**a**) PK1; (**b**) PK2. Adapted from [35], published by RSC, 2019. DOX—doxorubicin; Gly-Phe-Leu-Gly—glycine–phenylalanine–leucine-glycine linker; HPMA—hydroxypropyl methacrylate.

The first drug-delivery system developed for the PDEPT strategy consists of two components: (1) polymeric prodrug targeting the tumor by the effect of EPR, HPMA copolymer conjugated with doxorubicin (DOX) [31], and (2) copolymer HPMA conjugated with the cathepsin B enzyme, as an enzymatic trigger system. DOX was conjugated to the HPMA copolymer via a glycine–phenylalanine–leucine–glycine linker [32]. To enhance DOX concentration inside the cell and to ameliorate the release kinetics, a different mechanism for intratumoral release of the active drug was approached by exogenous administration of cathepsin B that accumulates in neoplastic tissue through the EPR effect. Cathepsin B is conjugated to the HPMA copolymer, which prevents degradation in the bloodstream [33,34]. Figure 3 shows the compounds PK1 and PK2, the first HPMA copolymers evaluated in clinical trials, PK1 conjugate contains DOX linked to the HPMA copolymer by a tetrapeptide sequence that is stable in the bloodstream, but sensitive to lysosomal hydrolysis. Therefore, the PK2 conjugate was added, which also contains *N*-acylated galactosamine-terminated side chains complementary to the asialoglycoprotein receptor located on hepatocytes [35].

Numerous PDCs have been obtained using synthetic polymers, such as HPMA, poly(ethylene glycol), poly(glutamic acid), poly(vinylpyrrolidone), poly(malic acid) or poly(vinyl alcohol) [36]. Most synthetic polymers have many disadvantages including toxicity, poor biocompatibility, and high cost of the production process [37]. To overcome these limitations, natural polymers have been exploited as safe alternatives in the preparation of drug-delivery systems. Natural polymers are biocompatible, biodegradable, and are available in large quantities as they are obtained from a great variety of renewable sources, like plants, animals, or microorganisms [38]. There are many types of natural polymers, such as proteins, polysaccharides, peptides, etc. [39]. Nanoparticles obtained from various natural polymers, especially polysaccharides, were developed as drug-delivery systems [40]. Polysaccharides possess many chemical functional groups which can be modified, thus increasing their potential applicability [41]. Moreover, some of them have the intrinsic ability to target specific receptors [42].

The aim of this study is the systematic presentation of biopolymers as potential carriers in antineoplastic prodrug therapy, focusing on their structure, characteristics, and the advantages they impress in comparison to conventional therapy. This article presents drug–polymer conjugates based on natural polymers such as chitosan (CTS), hyaluronic acid (HA), dextran (DEX), pullulan (PL), silk fibroin (SF), heparin (HEP), and *Auricularia auricula* polysaccharides (AAP).

## 2. Chitosan

Chitin is the most abundant natural aminopolysaccharide polymer, being the structural component that confers resistance to exoskeletons of crustaceans, insects, and the fungal cell wall. CTS is obtained by enzymatic or chemical deacetylation of chitin. It is a linear polysaccharide consisting of *N*-acetyl-2-amino-2-deoxy-D-glucose (*N*-acetyl-D-glucosamine) and 2-amino-2-deoxy-D-glucose (D-glucosamine) units [43]. Chitin and CTS are versatile biomaterials, similar in their properties to cellulose. Due to its solubility and the reactivity of free amino groups (-NH_2_), CTS is a useful material in various fields, having remarkable characteristics such as biocompatibility, low toxicity, biodegradability, film formation capacity, etc. [43,44,45,46].

**Figure 4:**
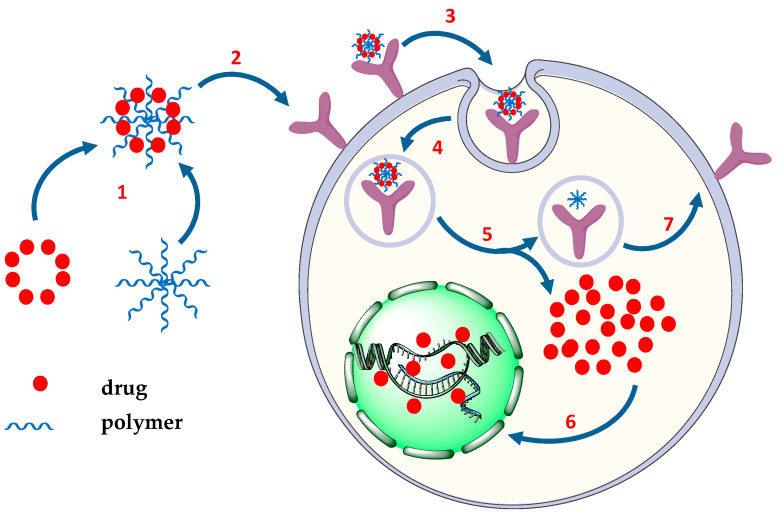
Mechanism of endocytosis of drug–polymer conjugates mediated by folate receptors. 1—drug–polymer conjugation; 2—FR binding; 3—membrane invagination; 4—endocytosis; 5—drug release; 6—DNA targeting; 7—FR externalization [69,70].

Nanoparticles (NPs) of folate-CTS conjugated with DOX and pyropheophorbide acid has been prepared using a tripolyphosphate-assisted ionotropic gelation method. CTS-containing NPs are hydrophilic, possess good biocompatibility and can improve membrane permeability. At the same time, folic acid (FA) functions as a ligand for targeting the cell membrane [47]. There is a specific receptor, namely the folate receptor (FR) which is a membrane protein with a high affinity for folate binding and transport. FR is overexpressed on the surface of neoplastic cells, in the ovaries, kidneys, colon, uterus, and lungs, thus becoming a potential target for tumor therapy [48,49,50]. The FR family is composed of three glycosylphosphatidylinositol-anchored membrane glycoproteins (FRα, FRβ and FRγ–identified in human tissues), rich in cysteine, capable of transporting drugs in the cytosol by endocytosis [51,52]. FR isoforms are considered promising therapeutic targets due to frequent overexpression of FRα in cancer cells of epithelial origin and FRβ in macrophages of tumors [53,54]. FRα has a high affinity for folates that are not usually included in food, becoming available for conjugates of FA with anticancer drugs [55]. The importance of the FR in the development and progression of cancer has determined the intensive study of FR-targeted therapeutic approaches [56]. Because FR transports folate through a high affinity endocytic pathway, a variety of antifolate drugs targeting FR and folate conjugates that use this transport mechanism, including cytotoxic drugs, have been developed [57,58,59]. The mechanism of endocytosis of folate–drug conjugates mediated by FR is schematically represented in Figure 4. The reduced folate or FA binds to FR and the receptor–conjugate complex is internalized by endocytosis, followed by the release of the drug [60,61,62,63]. To make the most of this effects for antitumor therapy, a series of compounds were developed among which: FA conjugated to macromolecules [64], NPs [65,66,67], and liposomes [68].

CTS in the form of water-soluble and biocompatible NPs, was conjugated with cytotoxic methotrexate molecules (MTX) by means of hydroxyl and amino functional groups [71]. CTS NPs have been attached to terbium and rare metal atoms as carriers for MTX. CTS functionalized luminescent rare earth doped terbium NPs as a drug-delivery system for MTX were studied. There has been a significant increase in the efficiency of CTS NPs loaded with MTX compared to the free drug [72].

Numerous biomaterials such as CTS-based nanoparticles have been recently developed for reasons related to the remarkable attributes of this natural polymer, as one of the most promising delivery vehicles for chemotherapy and cancer diagnosis, due to its unique characteristics: biodegradability, biocompatibility, enhanced penetrability of the cell membrane, high transport capacity of drug molecules, ability to have a multifunctional, and a prolonged circulation time [73].

The use of this natural polymer as a nanomaterial in the administration of drugs contributes to the improvement of the pharmacokinetic profile of the active drug molecule by increasing the cellular absorption of poorly soluble drugs [74,75]. It improves the bioavailability of the drug at effective doses.

Several studies have shown that the overall shape and appearance of NPs and their size contribute decisively in the distribution of the active drug molecule [76,77]. Balancing the size and geometry of NPs is essential for the efficiency of drug delivery, in order to achieve a prolonged systemic circulation time, thus improving their biodistribution and pharmacodynamics [78]. In the in vitro cytotoxicity assay, the positive charge of NPs has an important role in the uptake of the conjugate and in consequence of the active drug molecule into the tumor cells, demonstrating increased cell absorption and drug release into cells [79]. The absorption efficiency of these polymers is closely related to the properties of NPs (shape, particle size, hydrophobicity, and surface charges) [80,81,82,83]. NPs of polysaccharides also have excellent advantages in the transport of antitumor drugs [84,85,86].

Gemcitabine (GEM), a nucleoside analogue that is effective in a significant number of malignancies, has many limitations, such as reduced half-life leading to more frequent administration, low oral bioavailability, which limits the antineoplastic potential of this drug and increased toxicity [87]. Studies have been performed to design a vector in order to reduce the burden of frequent dosing and high toxicity associated with the use of GEM. Thus, NPCTS NPs encapsulating GEM have been developed and studied in vitro, ex vivo and in vivo [87,88]. Moreover, a peptide conjugate from CTS derivative, trimethyl CTS—and a cysteine-serine-lysine-serine-serine-aspartic acid-tyrosine-glutamine-cysteine peptide capable of improving the oral bioavailability of GEM as a consequence of its specificity to target intestinal cells and promote cell absorption have been developed [87].

Theranostics is a therapeutic strategy that uses a combination of a radioactive substance for diagnosis and an active therapeutic ingredient against neoplasms or metastases. Thus, theranostic agents are multifunctional agents composed of a payload carrier, therapeutic agents, and a ligand for targeting the complex [89,90]. The use of CTS as a theranostic agent in imaging investigations and it is based on its exceptional functional characteristics: positive charge at slightly acidic pH, biocompatibility, biodegradability, and low immunogenicity, which make it a multifunctional agent [91,92,93,94,95].

Thus, a complex of gold and CTS nanoclusters has been molded into theranostic NPs for bioimaging and suicide gene therapy [96]. Gold nanoclusters provided optical imaging properties. In addition, genes in the suicide-gene therapy strategy generate a bifunctional enzyme (cytosine deaminase-uracil phosphoribosyltransferase), which is involved in the transformation of the prodrug 5-fluorocytosine into the active cytotoxic molecule 5-fluorouracil (5-FU) in cervical neoplasm. In this case CTS stabilizes the theranostic NPs in optical emission. Structural investigations revealed the formation of the nanocomposite by binding gold atoms to the amino and hydroxyl groups of the CTS molecule, and the incorporation by encapsulation of the cytotoxic molecule of 5-FU was 96% [97].

In addition, studies have been performed to increase the efficiency of the 5-FU molecule coupled with the hyaluronidase (Hase) enzyme system incorporated into spherical CTS-NPs (5-FU-CTS-NPs) using three-dimensional (3D) spheroid culture HCT-116 (colorectal carcinoma cell line). Hase-loaded NPs (CTS-NPs) have been used in recent studies to enhance cancer treatment efficacy. It has been found that the use of the Hase enzyme increases the drug infusion in the tumor tissue. These CTS-NPs have been shown to increase the ability to deliver and enhance the anticancer activity of 5-FU in HCT-116 3D culture cell studies [98].

A CTS-based nanocomplex with lactobionic acid has been developed to target the sgVEGFR2/Cas9 plasmid and paclitaxel (PTX) as cytotoxic active molecule in the treatment of liver carcinoma. The study reported the tumor accumulation of the complex and the in vivo stability of the nanosystem. Furthermore, gene–drug loaded NPs promote the anti-tumorigenic pathway by suppressing pro-inflammatory cytokines (IL-6, IL-8) and protein expression in tumor angiogenesis (NF-κB p65), highlighting the potential of PTX when combined with gene therapy for overexpression of vascular endothelial growth factor 2 (VEGFR 2) on HCC cells, as a basis for synergistic gene–chemotherapy [99,100].

**Figure 5:**
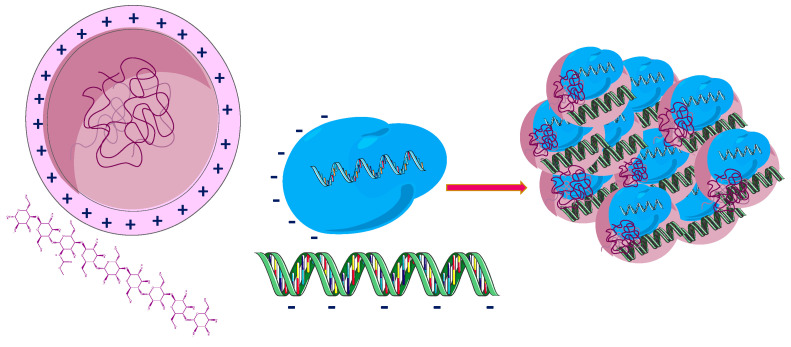
Nanoassembly of fluorescent protein−CTS Cas9/sgRNA and gene−donor. Adapted from [101], MDPI, 2020.

To date, there is a reduced number of studies on the use of CTS derivatives and other polymers for the delivery of genome editing components [101,102]. A CTS-negative fluorescent protein was encapsulated to deliver Cas9 protein and sgRNA. To obtain the Cas9 protein complex—CTS (Figure 5), polyglutamic acid was used to modify the protein. A 177 nm NP was formed by polyplexes composed of CTS, red fluorescent protein, a derivative of Cas9 protein and sgRNA specific for glutathione peroxidase-4 [101].

## 3. Hyaluronic Acid

Hyaluronic acid (HA) is a key compound of the extracellular environment, present primarily in the vitreous humor and in cartilage. It is a natural polysaccharide, first isolated in the 1930s from the vitreous humor of the bovine eye by Meyer and Palmer [103,104]. Structurally, it is a unique natural polysaccharide, with a linear structure of repeated disaccharide units, composed of D-glucuronic acid and *N*-acetyl-D-glucosamine [104,105].

HA has functional groups that can be used for different conjugations or can undergo various modifications, which makes this natural polymer a major component of multifunctional NPs useful in various therapeutic strategies [106,107,108,109,110].

As a consequence of its high compatibility, low toxicity, and biodegradability, HA is also extensively studied as a conjugating agent in PDEPT. In addition to these properties, HA can bond overexpressed receptors specific to tumor cells, so it can be used for targeting antineoplastic drugs. Therefore, HA has attracted a lot of attention as a vehicle for drug delivery [111]. HA has been shown to be an important carrier of drugs targeted to tumor cells. It shows important advantages such as:

- the use of lipid NPs with adequate HA coating as carriers of biocompatible drugs is an effective means of delivering the drug and at the same time significantly reduce side effects;
- improved distribution;
- improved release of drugs in cancer cells due to its high potential of targeted chemotherapy for tumors with increased CD44 receptor expression;
- enhanced efficacy [112,113,114].

At the molecular level, HA interacts with certain cell surface receptors, such as CD44, LYVE-1 (lymphatic vessel endothelium receptor-1), and RHAMM (receptor for hyaluronan-mediated motility), which are usually overexpressed in tumor tissue [110,115]. Following the linkage of HA to the CD44 receptor the internalization of this complex by endocytosis. HA is released and degraded by enzymatic reaction to low-molecular-weight components [109]. Thus, CD44 (an ubiquitous glycoprotein) has great potential to be an active target receptor as a consequence of overexpression in breast, colon, ovarian neoplasm, and squamous cell carcinoma [116]. HA is used to obtain conjugates for targeted drug delivery at specific sites due to its strong affinity for CD44. A series of advantages are expected following the conjugation of HA to cytotoxic agents regarding aqueous solubility, distribution, and efficacy [117].

**Figure 6:**
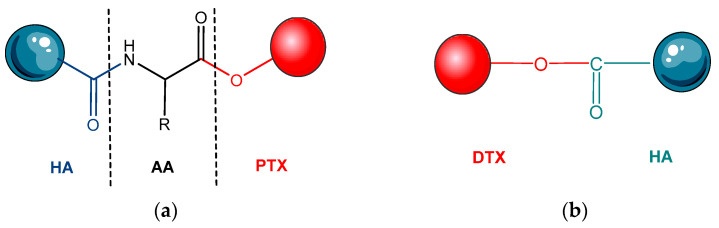

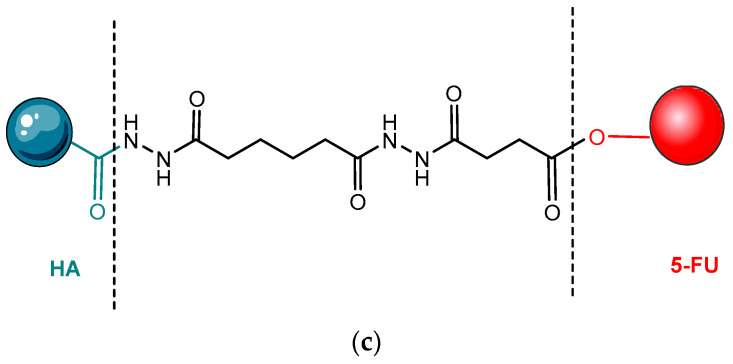
Drug–hyaluronic acid conjugates; (**a**) HA-amino acid-PTX conjugate [119]; (**b**) HA-DTX [125]; (**c**) HA-5-FU adapted after [129]. 5-FU—5-fluorouracil; AA—amino acid; DTX—docetaxel; HA—hyaluronic acid; PTX—paclitaxel.

Several developed HA conjugates are presented below:

Thus, a HA-AMINO ACID-PTX conjugate was studied. An amino acid acting as a crosslinker, or a linker was used in this conjugate: the carboxyl group of the amino acid is linked to the hydroxyl group of the cytotoxic drug molecule PTX and then the amino group of the amino acid was linked to the carboxyl group of HA to obtain a conjugate of hyaluronic acid-amino acid-PTX (Figure 6a) [118]. In the aqueous solution, the self-assembled amphiphilic conjugate into NPs and the active PTX molecule was surrounded by a hydrophilic HA structure. The active drug PTX was released by esterase mediated hydrolysis [119,120].

Numerous types of nanostructures have been studied, such as lipid-, carbon-, and polymeric-based NPs, that have been modified with HA to improve the delivery of cytotoxic molecules, such as DOX, to cancer cells [121]. A novel multifunctional system, that may be used for both tumor-targeting drug delivery and imaging, was developed through the assembly of a HA-DOX conjugate with a cationic conjugated polymer, represented by poly {[9,9-bis(6′-(*N*,*N*,*N*-diethylmethylammonium)hexyl)-2,7-fluorenylene ethynylene]-*alt*-*co*-[2,5-bis(3′-(*N*,*N*,*N*-diethylmethylammonium)-1′-oxapropyl)-1,4-phenylene]} tetraiodide (PFEP). In vitro release of the active molecule DOX, from the PFEP/HA-DOX complex, took place in the presence of hyaluronidase (Hase) within the first 15 min and reached a plateau after 25 min [35,122].

Another macromolecular conjugate, HA-Docetaxel (DTX), was developed with the aim of improving the pharmacokinetics and pharmacodynamics of DTX (a semisynthetic analogue of PTX). Drug release, cytotoxicity, cell absorption, cell cycle inhibition, and subacute toxicity were studied. In this sense, tumor cell lines that overexpressed CD44 receptors, such as human breast cancer cell lines MCF-7 and MDA-MB-231 were used [123,124]. Figure 6b shows a HA-DTX conjugate, containing a cleavable ester bond between the COOH group of HA and the 2′-OH group of DTX. Furthermore, the 2′-OH group in the PTX molecule (with a structure similar to DTX) has been found to bind to the COOH group in HA. 1-ethyl-3-(3-dimethylaminopropyl) carbodiimide/N-hydroxysuccinimide coupling chemistry was used for the activation of the COOH group of HA before conjugation. The obtained HA-DTX conjugate was characterized by spectral and thermal analysis [125].

HA-Cisplatin (*cis*-diamminedichloroplatinum (II), CDDP) is a conjugate that has been shown to reduce the significant side effects that limit the use of cisplatin. Through this conjugation there is an increase in the concentration of cisplatin in the lymphatic vessels, an increase in the distribution at the tumor site, a reduction in systemic toxicity and especially renal toxicity and there is an early inhibition of tumor metastases [126]. In order to efficiently deliver the CDDP-active molecule, especially in neoplastic ovarian tissue, titanium dioxide NPs have been designed as a nanometer-sized solid vector. TiO_2_ NPs were conjugated to HA, forming HA-TiO_2_ NPs that specifically target the ovarian cancer cells. HA-TiO_2_ NPs were loaded with CDDP to achieve a tumor-targeted drug-delivery system [127].

HA-5-FU is a conjugate in which the carboxylic group of HA binds to 5-FU through adipic acid dihydrazide and succinic anhydride linkers (Figure 6c). An increase in the antiproliferative activity of HA-5-FU conjugate was observed on various cancer cell lines [128,129].

**Figure 7:**
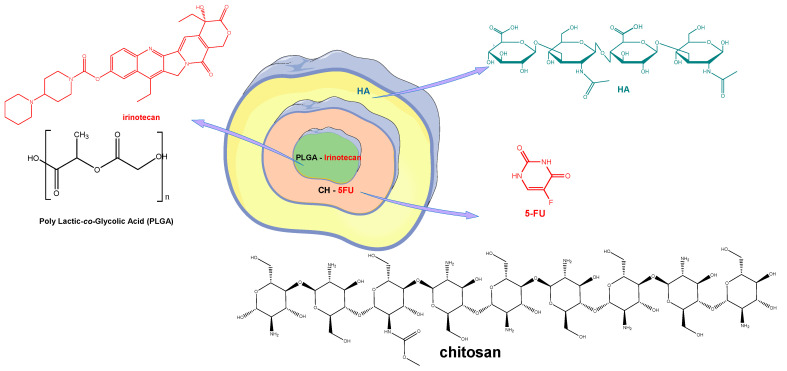
Irinotecan and 5-FU co-loaded nanoparticles [135]. 5-FU—5 fluorouracil; HA—hyaluronic acid; PLGA—poly(D,L-lactide-*co*-glycolide).

HA layer-by-layer NPs were developed in order to improve the efficacy of cytotoxic molecules and reduce their toxicity [130]. A new CTS-based HA hybrid polymer conjugate containing irinotecan and 5-FU was obtained in which irinotecan and poly(D,L-lactide-*co*-glycolide) represent the central core, CTS, and 5-FU the adjacent part and HA the outer layer (Figure 7). The in vitro and in vivo antiproliferative activity of the modified conjugate was superior to unmodified NPs, single-drug loaded NPs, or free drug, thus providing a promising strategy for targeted gastric cancer therapy [131,132,133,134]. Systems such as lipid–polymer hybrid NPs (LPNPs) with modified HA consisting of polymeric cores and lipid shells combine the advantages of polymeric NPs with those of lipid NPs or liposomes, increasing their physical stability and biocompatibility. Thus, HA modified, irinotecan and gene co-loaded LPNPs (HA-I/D-LPNP) were obtained and evaluated in vitro and in vivo on colorectal cancer cells and cancer-bearing mice. The conjugate system showed the highest antitumor activity and the best in vivo transfection efficiency, proving the beneficial effects of the targeted combination therapy [135].

## 4. Dextran

DEX is a natural polysaccharide studied as a polymeric carrier in targeted drug-delivery systems. From a structural point of view, DEX is a polysaccharide consisting of α-(1→6) and α-(1→3) glycosidic bonds of variable lengths (3–2000 kDa), which form a linear polymer through 1,6-glycosidic bonds with a certain degree of branching through 1,3-glycosidic bonds. The structure of DEX contains hydroxyl groups (OH) and terminal aldehyde groups (CHO), which can be chemically modified in order to obtain DEX-based biomaterials for various biomedical applications [136,137]. The hydroxyl groups are very important for conjugation with other substrates.

DEX in the form of NP microspheres has been used to improve the solubility of antitumor drugs and at the same time to improve their distribution at the target site of action [138,139]. DEX microspheres have many advantages, such as: biodegradability and biocompatibility, as well as non-immunogenicity and non-toxicity, all important factors for clinical applications. Regarding their physico-chemical profile, these microspheres are advantageous because they are easy to filter, are neutral, and soluble in water. Also, the functional groups of DEX provide promising advantages for biological and imaging applications [140,141,142,143]. Thus, a controlled-release system based on DEX microspheres has been developed, the vehicle of choice for the administration of mitomycin C, a potent promoter agent, which works by bioreductive activation in antineoplastic therapy [144].

Stimuli-sensitive covalent PDCs are promising alternatives to polymer NPs that rely on physical drug encapsulation, due to their advantage of providing much more precise control over the active drug dosage and release [145,146,147]. The unique ability of PCDs to self-assemble into NPs and stimuli-sensitive drug release are among the advantages of this NP-based drug-delivery systems [23,145,148,149]. 

**Figure 8:**
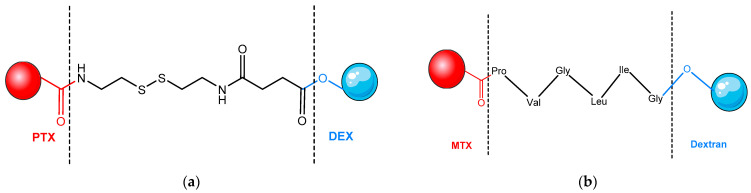
Dextran conjugates: (**a**) DEX–PTX conjugate [151] and (**b**) DEX–MTX conjugate [152]. DEX—dextran; Gly—glycine; Ile—isoleucine; Leu—leucine; MTX—methotrexate; Pro—proline; PTX—paclitaxel; Val—valine.

The conjugate between DEX and PTX through a disulfide linker is presented in Figure 8a. DEX-S-S-PTX PDC demonstrated significant cytotoxicity on BT-549 and MCF-7 tumor cells, with the release of PTX in response to the intracellular reducing medium of tumor cells. DEX-S-S-PTX NPs showed IC50 (half-maximal inhibitory concentration) values and mechanism of action similar to those of free PTX, emphasizing the intracellular release of the drug [150,151].

Due to its hydrophilic, biocompatible, and biodegradable properties, DEX has been used to obtain a conjugate with another cytotoxic drug, such as MTX. MTX is linked to DEX by a peptide that can be cleaved by matrix-metalloproteinase-2 (MMP-2) and matrix-metalloproteinase-9 (MMP-9), two important enzymes present in tumor tissue. Its antitumor efficacy and systemic side effects were evaluated in vivo and were compared with free MTX and a similar conjugate with a different linker, insensitive to MMP enzymes, in doses equivalent to intraperitoneal administration, that have been released in tumor tissue and not in a healthy tissue. This study showed that DEX-MTX conjugate has been effective in releasing the drug into tumors that express the specific enzymes MMP-2 and MMP-9, which are characteristic of malignant tumors. The linker used is a peptide structure consisting of the following amino acids: glycine–isoleucine–valine–glycine–proline–leucine [152] (Figure 8b).

The in vitro and in vivo antitumor activity of DOX encapsulated NPs using deoxycholic acid (DA) conjugated dextran (DEX-DA) was evaluated on CT26 colon cancer cell line. The DEX-DA nanoparticles on which the DOX molecules have been incorporated had spherical shapes and particle sizes between 50–200 nm. In addition, the rate of drug release was accelerated in acidic pH compared to the alkaline pH. The results of the cytotoxicity assay using CT26 cell line, showed that the NPs possessed increased antitumor activity compared to free DOX [153].

Another approach for targeting the active DOX molecule is in the form of NP in the DEX-FA polymer conjugate (DEX-FA-NPs). The FR-mediated endocytosis and in vivo targeted delivery have been demonstrated for DEX-FA-NP, with an increased antitumor activity and reduced side effects [154].

**Figure 9:**
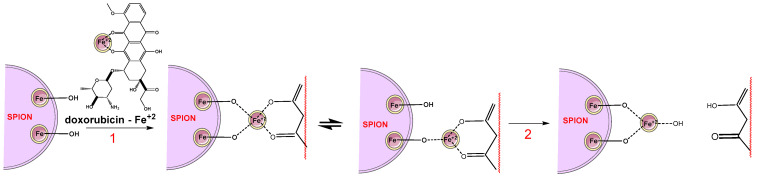
DSPION nanoparticles with dextran and doxorubicin: (1)—DOX-Fe^2+^ loading; (2)—DOX releasing. Adapted from [158], Elsevier, 2008.

Another approach is that of magnetic NPs combined with different materials for various magnetically guided drug-delivery systems that can be used for targeted administration of cytotoxic drugs. For example, transferrin conjugated magnetic DEX-spermine NP (DS-NP) was developed for blood brain barrier penetration of drugs [155]. On the other hand, DEX-coated superparamagnetic iron oxide NPs (DSPIONs) have been developed as carriers for cytotoxic molecules due to their biocompatibility and ability to reduce the toxicity of some cytotoxic molecules, such as DOX [156,157]. Figure 9 presents the loading of polymer and DOX releasing from ferrofluids. In order to avoid the DOX oxidation, Fe^2+^ was used [158].

Other strategies for obtaining NP conjugate systems with different pH-sensitive cytotoxic molecules have been approached [159,160,161]. An example is represented by the covalent conjugation of oxidized DEX (oDex) with two cytotoxic molecules, Pt (IV) prodrug and DOX, with the formation of oDex-Pt + DOX NPs with an average diameter of approximately 180 nm, characterized by a pH and reduction dual sensitivity. The efficient cellular absorption of oDex-Pt + DOX NPs in human cervical carcinoma HeLa cells was identified using confocal laser scanning microscopy. The antitumor activity of oDex-Pt + DOX NPs was similar to the combination of the free drugs, but the oDex-Pt + DOX NPs displayed an improved ability to reverse tumor resistance in cisplatin-resistant A549 lung cancer cells [162,163].

## 5. Pullulan

PL is a natural polysaccharide, an exopolysaccharide, composed of maltotriose units, biocompatible biopolymer, biodegradable, non-toxic, nonmutagenic, and noncarcinogenic [164]. It is a product of aerobic metabolism of a species of fungus *Aureobasidium pullulans* [165]. Structurally, this linear natural polymer consists of three units of glucose, linked by an α-1,4 glycosidic bond, the units of maltotriose thus formed being linked by an α-1,6 glycosidic bond. PL is obtained naturally from starch under the action of the polymorphic fungal species *Aureobasidium pullulans* [166,167].

This polysaccharide is extensively studied for its multifunctional use in systems with various biological activities such as antimicrobial, antiproliferative, anti-inflammatory, antioxidant, immunomodulatory, etc. All studies have shown the versatility of pullulan-based delivery systems in the form of NPs, microparticles, hydrogels, and micelles. The in vivo and in vitro evaluation of the use of pullulan in conjugation with cytotoxic molecules and the release of drugs from PL conjugates has shown promising results [168].

Low immunogenicity and satisfactory solubility in aqueous solvents have led to its use as a polymer for drug delivery in antineoplastic therapy. There is extensive research for these uses of PL, including in NP formulations, which demonstrates its flexibility and relevance as a biomaterial [169,170]. PL NPs conjugated with various cytotoxic molecules generate minimal side effects compared to the use of these drugs alone and achieve sustained or prolonged delivery to targeted tissues [171]. The special properties of polysaccharides in the form of NPs, in terms of the ability to locate tumor cells is a major advantage for obtaining a remarkable therapeutic efficacy in vivo [172,173,174].

Pullulan as a polymer for conjugation with various cytotoxic active molecules is studied as a vehicle for the targeted delivery of these molecules to various organs: liver, spleen, lungs, brain, etc., and for sustained or prolonged release of certain cytotoxic molecules to the specific target site [175,176,177].

Combination chemotherapy becomes necessary when chemoresistance to some cytotoxic drugs limits the curative effect. Thus, in order to overcome these inconveniences, different derivatizations were used to obtain PL-DOX-PDTC or PL-DOX-S conjugates. For the PL-DOX-PDTC, a pyrrolidine dithiocarbamate (PDTC) inhibitor of the nuclear factor kappa B was added to the pharmaceutical formulation [178], and for PL-DOX-S, sorafenib (S) an inhibitor of the enzyme tyrosine protein kinase was added [179].

**Figure 10:**
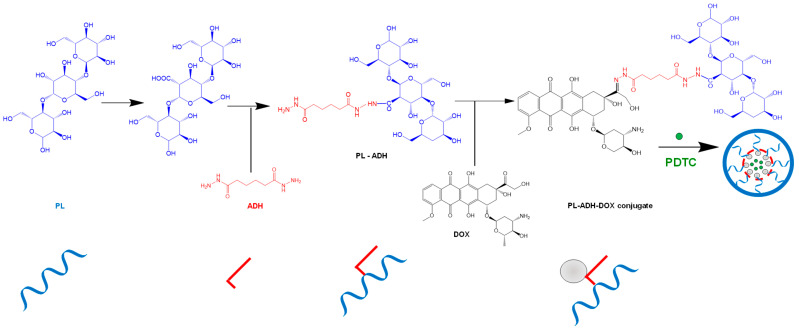
Doxorubicin–PDTC co-loaded nanoparticles. Adapted from [178], published by J Mater Chem B, 2015. ADH—adipohydrazide; DOX—doxorubicin; PDTC—pyrrolidine dithiocarbamate; PL—pullulan.

PL-DOX-PDTC nanoparticles are delivery systems that have been evaluated in hepatocellular and murine breast carcinoma models. NPs synthesized from pullulan-DOX-pyrrolidine dithiocarbamate had a corresponding stability at pH 7.4. Adipohydrazine showed a higher drug loading capacity and a better biocompatibility compared to hydrazine hydrate [178] (Figure 10).

**Figure 11:**
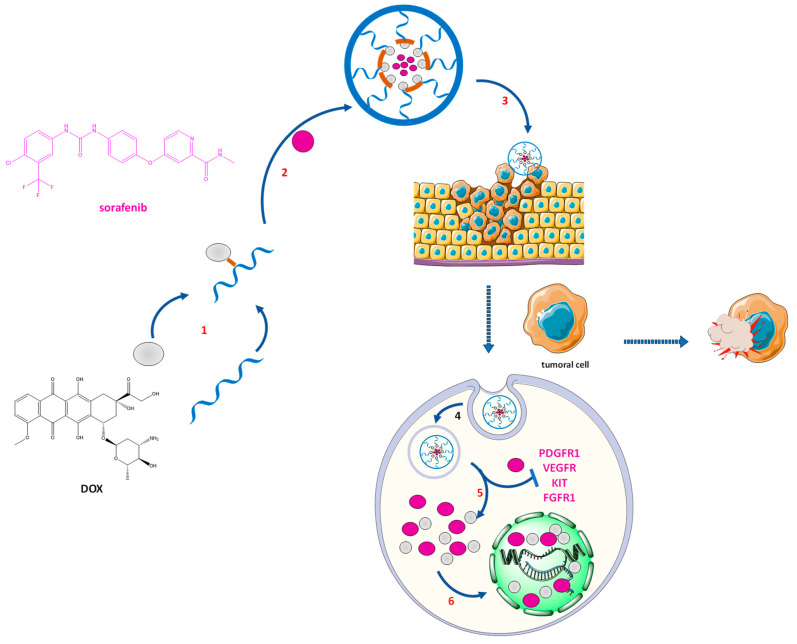
Doxorubicin–sorafenib co-loaded nanoparticles and their synergic mechanism of action [179,182] DOX—doxorubicin; FGFR1—fibroblast growth factor receptors; KIT—tyrosine-protein kinase; PDGFR1—platelet-derived growth factor receptors; PL—pullulan; VEGFR—vascular endothelial growth factor. 1—doxorubicin-pullulan conjugation; 2—self-assembly of the NP with sorafenib; 3—membrane invagination; 4—endocytosis; 5—drug release; 6—DNA targeting.

pH-sensitive NPs were obtained by encapsulation of sorafenib, a water-insoluble molecule, in the PL-DOX complex [180] (Figure 11). Synergistic antiproliferative activity has been investigated on 4T1 tumor cell lines [179,181].

Lately, many conjugates were synthetized between modified PL and cytotoxic molecules, which were actively directed to certain tumor tissues. For example, some compounds were obtained by conjugating carboxymethyl pullulan (CM-PL) and DOX. Thus, an improved anticancer efficacy in various tumor cells and reduced systemic toxicity were observed for DOX-peptide-CM-PL conjugates [183]. Another study reported reduced side effects of DOX conjugated with CM-PL used in liver cancer [184].

The structural modulations required to derivatize the pullulan molecule are very different, and include reactions of: etherification, esterification, amidation, oxidation, sulfation, and copolymerization by replacement of hydroxyl groups [185,186,187,188]. Pullulan NPs, nanogels, microspheres, and mycelium, due to their improved permeability and retention effect, act as efficient drug-delivery systems [189]. The active drug molecules conjugated with this polymer in the form of NPs accumulate more in cancer cells than in normal cells, thus decreasing the toxicity of drugs to normal cells [190,191,192].

Moreover, amphiphilic polymers with hydrophobic groups, such as cholesterol (CHS) groups, were obtained through esterification. The CHS-modified PL with a hydrophobic core [192], and cholesteryl-modified aminated PL NPs were also obtained [193].

**Figure 12:**
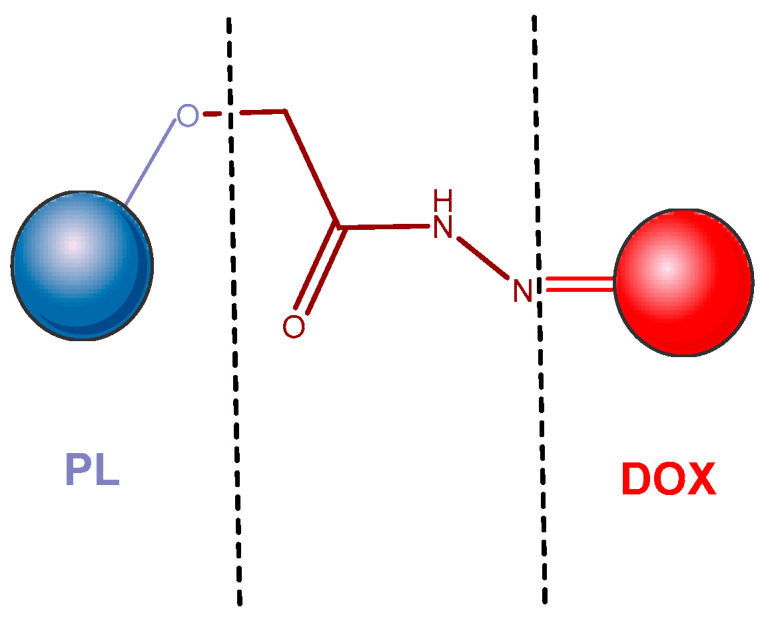
Pullulan–doxorubicin conjugate [194]. DOX—doxorubicin; PL—pullulan.

A hydrazone bond, that is stable in a neutral medium, but easily cleavable in a slightly acidic environment was used to attach DOX to the pullulan polymer, thus obtaining a pH-sensitive pullulan-DOX conjugate (Figure 12). In aqueous solution, the conjugates spontaneously self-assembled into structured NPs with DOX in the center and pullulan as the outer shell. The NP diameter varies depending on the drug loading degree, being between 50 and 110 nm. The pH-dependent release of the active drug has been demonstrated in vitro: in 2 h, over 75% DOX was released at pH 5.0, and about 15% DOX was released at pH 7.4. This supports the rapid diffusion of DOX into liver tumor cells by actively targeting NP pullulan-DOX, without the need for the introduction of an additional ligand [194].

Some studies highlighted the improvement of the half-life and increase of plasma concentration of the epirubicin, when it was delivered by a CHS-PL system in liver, heart, or renal cancer cells [195,196,197]. Mitoxantrone delivered from CHS-substituted pullulan large-sized NPs presented a high bioavailability and growth inhibition of bladder cells [198]. An increased releasing of the DTX from a CHS-pullulan conjugate (~67%) after cleavage of the hydrazone bond was observed and the conjugate could have a therapeutic potential in breast cancer [199]. CHS-PL-DTX inhibited the growth of tumor cells in a lung cancer mouse model [200].

Several synthetized PL-acetate (PLA) conjugates presented different advantages such as: (i) NPs with PLA and epirubicin displayed increased drug release and cytotoxicity against KB nasopharyngeal epidermal cancer cells [201,202]; (ii) PLA-DOX conjugates presented an improved drug cytotoxicity to the MCF-7 tumor breast cell line [203]; and (iii) PTX and all-trans retinoic acid conjugated with PA presented a synergistic antiproliferative effect on CT26 colon carcinoma cells [204].

Also, a succinylated PL conjugated with cisplatin was obtained. It had an increased inhibitory effect on hepatocellular carcinoma cells compared to the effect on lung epithelial cells [205].

A thermo-responsive nanogel containing a conjugate of DOX (pullulan-g-poly(L-lactide)-DOX) presented an enhanced release of the antitumoral drug [206]. NPs containing DOX conjugated with folate-decorated maleilated pullulan (FA-MP) presented an increased cell absorption, targeting capacity, and cytotoxicity in A2780 ovarian cancer cells compared to the free DOX [207]. Also, NPs with DOX incorporated in FA-conjugated pullulan/poly(D,L-lactide-*co*-glycolide) graft copolymer NPs presented a better targeting capacity of the folate receptor in tumor cells [208].

The PL stabilized gold NPs were coupled with 5-FU and FA. The in vitro and cytotoxicity studies confirmed the potential of these NPs as an alternative carrier for targeting liver cancer cells with a distribution of 55% in liver tissue 2 h after administration [209].

In an experimental model, a drug carrier system based on MTX and combretastatin A4 loaded on pullulan used as combined therapy against hepatocellular carcinoma prolonged systemic circulation and presented an increased antiproliferative activity [210].

PTX was loaded on core-crosslinked pullulan and lipoic acid NPs [211]. Lipoic acid is a potent antioxidant beneficial in metabolic syndrome [212], with a very good safety profile [213]. It contains a disulfide bond in the ring that is reduced to thiol groups [213], with a significant capacity of polymerization [211]. The results of the cellular studies performed on liver cancer cells exhibited great cytotoxicity, an increased systemic retention time and reduced plasma clearance in the cell line [211].

The drug-delivery system based on *O*-urocanyl pullulan improved DOX accumulation and retention in the MCF 7 breast cancer cell line. Approximately 72.1% of the drug was released within 24 h of administration [214].

Acetylated pullulan and low-molecular-weight polyethyleneimine were conjugated in order to obtain a degradable cationic nanogel. This nanogel was coated with HA and it presented an enhanced tumor penetration due to the necrosis induced by itself and implicitly by the increasing of the paracellular transport. This facts permitted the releasing of the PTX in a large and deep tumoral region [215].

## 6. Heparin 

**Figure 13:**
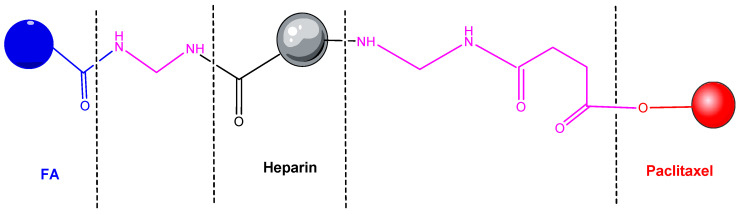
Heparin–folic acid–PTX conjugate [216].

HEP is a natural polymer and could be considered an alternative for drug delivery in cancer cells. A HEP-FA-PTX conjugate was synthetized. In this conjugate, PTX is attached by covalent bond to FA and HEP (Figure 13). It was used in experiments on tumor xenografts of human cells cultured subcutaneously or on laboratory animals [216]. This polymeric conjugate can self-assemble into spherical mycelium in the aqueous medium by binding PTX to HEP by hydroxyl grouping and a pH sensitive linker. Cytotoxicity tests have shown that this conjugate possesses significant cytotoxicity against MDA-MB-231 tumor cells, and FA enhances the targeting of the compound [217]. Cell absorption and intracellular distribution was studied by confocal laser scanning microscopy (CLSM) [218].

HEP grafted with cysteine was loaded with poorly water-soluble chlorambucil for obtaining an amphiphilic polysaccharide-drug conjugate. NPs were biocompatible with HaCaT normal cells and had high absorption in HeLa tumor cells [219]. HEP-pluronic conjugate encapsulated 5-FU and hydrated cisplatin. The obtained complex has high drug-loading capacity, slow and sustained release, Hep-F127 cytocompatibility, and a significantly antiproliferative effect on NCI-H460 lung cancer cells [220]. Also, HEP could be used for obtaining a nanogel in combination with poloxamer 403. Due to their synergistic effect, curcuminoid, and cisplatin hydrate co-loaded on this nanogel present an increased survival time and an in vivo antitumor activity in breast cancer. Moreover, undesired consequences of cisplatin were decreased. All these suggested a targeted in tumor site delivery [221]. On the other hand, dalteparin, a low molecular weight HEP with antitumor and antiangiogenic activity, was conjugated with poloxamer 407. Subsequently, this co-polymer was used for the addition of a synthetic nanosilicate (laponite RDS) loaded with DOX. The in vivo and in vitro studies highlighted the synergic action of HEP and DOX, and an increased antitumor efficacy evaluated after a single administration on xenograft S180 sarcoma [222].

## 7. *Auricularia auricula* Polysaccharides

**Figure 14:**
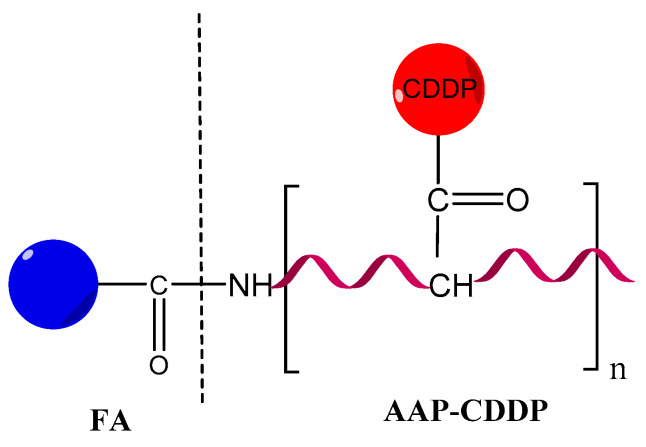
FA-AAP-CDDP polymeric complex.

A promising drug-delivery system based on a polysaccharide biopolymer was isolated from the medicinal fungus *Auricularia auricula*. An experimental study concluded that a lectin containing four peptides inhibited the A549 cells proliferation by regulating the expression of some cancer-related genes [223], such as JUN an oncogenic transcription factor [224], TLR4 (toll-like receptor 4) expressed on immune cells and also on tumor cells [225], and MYD88 (myeloid differentiation factor 88) that promotes colorectal cancer cells [226]. NPs containing polysaccharide polymers from *Auricularia auricula* (AAP) and CTS were very efficient for DOX entrapping and penetrating in tumor cells. Thus, AAP represents a promising option as an antineoplastic drug carrier [227]. An APP modified with histidine (His-AAP) was loaded with PTX and the His-AAP-PTX conjugate efficacy was tested on mouse model and showed the inhibition of tumor cells proliferation and the reduction of systemic side effects [228]. Another type of AAP conjugate containing FA and *cis*-diamine dichloroplatinum (CDDP), with favorable effects in cervical cancer was developed [229,230]. It has been shown to increase the efficiency of cisplatin and to reduce side effects. The FA-AAP-CDDP complex (Figure 14) may induce the production of cytokines such as interleukin-2, interleukin-4, and interferon in laboratory experiments on mice. The FA-AAP-CDDP complex also promoted the expression of Bax and caspase-3 proteins, activated the mitochondrial apoptotic pathway of tumor cells, and had a higher intratumoral accumulation [231].

## 8. Protein-Drug Conjugates and Peptide-Drug Conjugates

For targeted therapy various drug-delivery systems are conjugated with proteins or peptides. In this case cytotoxic agent (payload) is connected to the tumor-targeting carrier via a cleavable (stimuli responsive) or non-cleavable linker. Tumor-targeting carrier may be an antibody, a protein, or a peptide and the resulting conjugates are antibody–drug conjugates (ADCs), protein–drug conjugates (PrDCs) and peptide–drug conjugates (PeDCs). A key factor, in the design of all these conjugates is the linker which influences the stability of the conjugate, modulates the release of the cytotoxic agent and the pharmacokinetics [232,233,234,235,236].

ADCs with humanized antibodies showed low immunogenicity and better half-lives in comparison with ones with murine antibodies, but due to size and complex structure their use is somehow limited to hematologic malignancies and rarely to solid tumors. By means of PEGylation certain properties of anti-bodies can be modified (increased half-lives and reduced immunogenicity). Nowadays there are twelve ADCs approved by FDA (starting with Gemtuzumab ozogamicin, more than two decades ago) [236,237,238,239].

PrDCs ensures improved pharmacokinetics, long blood residence and reduced toxicity by selective distribution in targeted cells. Physicochemical criteria are used to identify suitable proteins for conjugation. Several PrDCs (with albumin, transferrin, gelatin, hemoglobin, fibrinogen, insulin, etc.,) are now in preclinical or clinical phases of clinical trials [236,240,241,242,243,244].

Some limitations of ADCs and PrDCs may be avoided by using PeDCs. The synthesis of peptides is cheap and structural modifications can be easily performed to optimize stability, bioavailability, tumor targeting, cellular uptake, etc., PeDCs have smaller molecular weight in comparison with ADCs and PrDCs, which makes them more suitable for the treatment of solid tumors compared to ADCs and PrDCs. Limitations of PeDCs are related to renal clearance, reduced stability, short half-lives, and nonspecific cell uptake (in case of cell-penetrating peptides). Some of the limitations may be overcome by peptide cyclization, replacement of L-amino acids with D-amino acids, PEGylation, etc. On the market, there are only two FDA approved PeDCs: LUTATHERA^®^ (lutetium Lu 177 dotatate) and Pepaxto^®^ (melphalan flufenamide). The marketing of the latest one is currently discontinued in US [235,242,245,246,247,248].

### 8.1. Silk Fibroin

As presented, natural polymers have many advantages, the most representative being those related to high biocompatibility and the ability of structural modulation that offers versatility as drug delivery vehicles through specific interactions with some biomolecules. These polymers are generally used for conjugation with small molecules, proteins and DNA in various biomedical applications, such as wound healing or anticancer therapy [249].

Silk fibroin (SF) produced from the cocoon of the *Bombyx mori* silkworm is widely used and has various biomedical applications, including the controlled release of drugs. Silk fiber derived from other insect species has also been investigated. Silk fibroin consists mainly of fibroin and sericin [250,251,252].

SF is a high-molecular-weight protein, being characterized by a good biocompatibility, biodegradability, and high mechanical and tensile strength [253,254,255,256,257,258,259]. Various techniques such as freeze-drying and physical or chemical crosslinking, centrifugal coating, and electroplating are used to obtain a variety of SF-based materials, including films and hydrogels [257,258,259].

SF has many biomedical applications, including cancer therapy, tissue engineering, controlled drug release, and bone and skin tissue regeneration [260,261]. Its structure consists of a heavy chain (with a molecular weight of 391.6 kDa) and a light chain, that are linked through a disulfide bond at the C-terminus [262,263]. The heavy chain contains 12 crystallizable hydrophobic domains and 11 more hydrophilic amorphous domains, making it an amphiphilic copolymer [264]. The β structures of the crystallizable domains, formed by the repetition of the glycine–alanine–glycine–alanine–glycine–serine amino acids chain, confers high thermal stability and excellent mechanical properties [265].

Silk-based delivery systems have excellent properties and can be used to deliver many therapeutic substances for cancer treatment, such as: (i) chemotherapeutics, (ii) nucleic acids, peptides or proteins, (iii) inorganic compounds, (iv) photosensitive molecules, and (v) plant derivatives. It has been reported that intracellular degradation of SF NPs is dependent on lysosomal enzymatic function. Therefore, these SF-based nanocarriers perform a critical function, represented by the degradation of the release system, and can be considered a safe in vivo drug-delivery systems [251,266].

SF and its derivatives obtained through various structural modulations that can lead to other self-assembling NPs, have great potential in the delivery of genes, small molecules and proteins [267,268,269].

Thus, for example, a functionalized SF complex was developed using FA, in carbon nanotubes in the form of hydrogel, to obtain a system for directing the active molecule of DOX in tumor tissue [257,270].

Several anticancer molecules conjugated with SF have shown significant potential in preliminary studies on various cancer cell lines amongst which bone cancer cells—DOX and breast cancer cells–tamoxifen, DTX, and DOX. Also in vivo trials were performed in mice showing superior effects of GEM loaded NPs [271,272,273].

There are currently several studies proving the efficacy of SF-*B. mori*, SF, and SELP (silk-elastin-like polymer) release systems incorporating chemotherapeutic substances. Thus, for example, the active pharmaceutical ingredients that can be incorporated into *B. mori* SF and SELP-based delivery systems include: (i) DOX with therapeutic indication in breast cancer, cervical cancer, neuroblastoma; (ii) PTX with therapeutic action in gastric, pancreatic, cervical or breast cancer; (iii) cisplatin used in lung or ovarian cancer; (iv) 5-FU for gastric and breast cancer; (v) floxuridine with therapeutic indication in cervical cancer; (vi) MTX for breast cancer; and (vii) GEM for lung cancer [251,274].

On the other hand, there are some examples of herbal derivatives with the role of therapeutic agent incorporated in SF release systems, such as [251,274]:

- curcumin with therapeutic indication in liver, colorectal and breast cancer;
- resveratrol with therapeutic indication in colon cancer;
- triptolide/celastrol with therapeutic indication in pancreatic cancer;
- emodin with therapeutic indication in breast cancer;
- α-mangosteen with therapeutic indication in colon and breast cancer, etc.

### 8.2. Centyrins

Centyrins (CTRs) are non-antibody, small-size proteins that are engineered from the human protein Tanascin C (found in extracellular matrix). There are some CTRs’ properties (the lack of disulfide bonds, the small molecular size, increased stability, low immunogenicity, improved tissue penetration, simple drug conjugation, etc.,) which make these molecules the perfect candidates for targeted delivery applications. CTRs can be conjugated with small interfering ribonucleic acids and an increase in efficiency of gene target modulation was observed [275,276,277].

The particularities of the polymer–cytotoxic drug conjugates presented in this study are summarized in Table 1.

One important approach in cancer treatment is chemotherapy. Unfortunately, chemotherapeutic agents often possess low bioavailability, poor solubility, short half-life, lack of specificity, and may induce multi-drug resistance. Biopolymers and biopolymer-nanomaterials are perfect candidates for conjugation with drugs, in order to perform effective targeted therapy. Conjugates confer improved stability of cytotoxic agent, possesses improved absorption and bioavailability and are able to reduce toxicity in healthy cells [278].

Current progress in both polymer science and bioengineering allows the design and synthesis of smart polymers which are able to release the drug under appropriate stimulus (stimuli-sensitive polymers): temperature, pH, ultrasound, pH, ionic strength, enzymes, biomolecules, etc.) [279,280,281,282].

## 9. Conclusions

The targeted therapy strategies in which the penetration of cancer cells and the retention of the drug are considerably improved and are accomplished by delivery systems. Thus, the active drug molecule can bond to various target sites on cancer cells. These antitumor strategies aim to increase efficacy and decrease the adverse effects of conventional chemotherapy, and the use of polymeric conjugates contributes to overcoming physicochemical and pharmacological limitations, thus providing increased water solubility, half-life, and therapeutic index of drugs, as well as protection and reduced toxicity. Many delivery systems present conjugates between antitumoral drugs and polysaccharides, peptides or proteins that are conjugated directly or by means of stimuli-sensitive linkers (i.e., sensitive to the action of enzymes or to pH). This study was conducted on several natural polymers that are frequently mentioned in the scientific literature, like: chitosan, hyaluronic acid, dextran, pullulan, heparin, *Auricularia auricula* polysaccharides, silk fibroin, and centyrin. Conjugates comprising these natural polymers can be used in therapy, but further studies should be conducted in order to assess their safety and therapeutic efficacy.

## Figures and Tables

**Table 1:** The summarizing of particularities of polymer–cytotoxic drug conjugates.

Polymer	Antitumoral Drug (s)	Particularities	References
**Chitosan**	DOX	NPs of folate-CTS were conjugated with DOX and pyropheophorbide acid	[47]
	MTX	NPs incorporated MTX and an amino acid- with pH-responsive properties	[71]
	MTX	CTS functionalized luminescent rare earth doped terbium NPs presented an increased efficiency	[72]
	GEM	The binding by CSKSSDYQC peptide improved the oral bioavailability of GEM	[87]
	5-FU	Gold nanoclusters presented a high-capacity of -FU incorporation	[97]
	5-FU	Hase-loaded NPs enhanced the efficacy of chemotherapeutic drugs	[98]
	PTX	Lactobionic acid functionalized and stimuli-responsive CTS based nanocomplex were used to co-deliver sgVEGFR2/Cas9 plasmid and PTX	[100]
**HA**	PTX	The cross-linkage of PTX to HA was realized with an amino acid	[118]
	DOX	Advanced HA nanostructure based on PFEP improved DOX delivery in tumour cells in the presence of Hase	[122]
	DTX	Nanoliposomes HA-DTX had an enhanced delivery of DTX after activation of COOH group of HA by 1-Ethyl-3-[3-(dimethylamino)-propyl]-carbodiimide hydrochloride and N-Hydroxy succinimide coupling chemistry	[125]
	Cisplatin	The chloride ligand in coordinating platinum made possible the drug liberation from the complex	[126]
	Cisplatin	HA-TiO_2_ NPs specifically targeted ovarian cancer cells	[127]
	5-FU	Adipic acid dihydrazide and succinic anhydride linkers were used for 5-FU binding to HA	[128,129]
	Irinotecan and 5-FU	Layer-by-layer NPs containing Irinotecan and 5-FU conjugated with HA, CTS, and poly(D,L-lactide-co-glycolide) presented a superior antiproliferative activity	[135]
**DEX**	PTX	The DEX-PTX conjugate through a disulfide linker had a significant cytotoxicity	[150,151]
	MTX	MTX was linked to DEX by a Gly-Ile-Val-Gly-Pro-Leu peptide cleaved by MMP-2 and MMP-9 from tumor tissue	[152]
	DOX	The release of DOX form DEX-DA NPs was accelerated in acidic pH	[153]
	DOX	The targeted delivery of DOX from DEX-FA NPs was increased and FR-mediated	[153]
	DOX	DEX-coated superparamagnetic iron oxide NPs were used as DOX carriers for cytotoxic molecules	[158]
	Pt (IV) and DOX	Pt (IV) and DOX from NPs with oDEX conjugated, had an improved ability to reverse tumour resistance in cisplatin-resistant in A549 lung cancer cells	[163]
**PL**	DOX and PDTC	Adipohydrazine improved the biocompatibility and the capacity of drug loading in PL-DOX-PDTC NPs	[178]
	DOX and S	PL-DOX-S conjugate had a synergistic antiproliferative activity	[179,180,181]
	DOX	DOX-peptide-CM-PL conjugate had an improved anticancer efficacy and reduced systemic toxicity	[183,184]
	DOX	The releasing of DOX from PL-DOX NPs was pH-dependent	[194]
	Epirubicin	The epirubicin half-life and plasma concentration was increased in the CHS-PL-epirubicin system	[195,196,197]
	Mitoxantrone	Large-sized NPs based on CHS-substituted pullulan presented high bioavailability and growth inhibition of cancer cells	[198]
	DTX	A hydrazone bond was used for the conjugation of CHS-PL with DTX which increased the releasing and antitumor activity of DTX	[199,200]
	Epirubicin, DOX, PTX-all trans retinoic acid	NPs based on PL-acetate increased the cytotoxicity of the drug	[201,202,204]
	Cisplatin	The increased antitumoral effect of cisplatin in succinylated PL conjugates was observed	[205]
	DOX	NPs with DOX and pullulan-g-poly(L-lactide), FA-MP, and FA-conjugated pullulan/poly(D,L-lactide-*co*-glycolide) presented an enhanced releasing of DOX	[206,207,208]
	DOX	The potential of PL stabilized gold NPs containing DOX and coupled with 5-FU and FA was observed	[209]
	MTX and cambrestatin	The antiproliferative activity of MTX and combretastatin conjugated with PL was noted	[210]
	PTX	PL and lipoic acid NPs containing PTX had a great antitumor capacity	[211]
	DOX	*O*-urocanyl pullulan improved DOX in a breast cancer cell line	[214]
	PTX	A cationic nanogel based on acetylated pullulan, low molecular weight polyethyleneimine, and HA improved the tumor penetration of PTX	[215]
**HEP**	FA and PTX	PTX conjugated with HEP through a pH sensitive linker and a hydroxyl grouping presented a significant cytotoxicity against MDA-MB-231	[217]
	Clorambucil	HEP grafted with cysteine improved the biocompatibility and absorption of clorambucil	[219]
	Cisplatin and 5-FU	Cisplatin conjugated with HEP-pluronic and 5-FU presented a significantly antiproliferative effect on lung cancer cells	[220]
	Curcuminoid and cisplatin hydrate	Curcuminoid and cisplatin loaded on poloxamer 403 had a synergistic in vivo antitumor effect	[221]
	DOX	DOX loaded on dalteparin, poloxamer 407 and laponite RDS had an increased antitumor efficacy on xenograft S180 sarcoma	[222]
**AAP**	DOX	AAP and CTS based NPs were efficient for entrapping DOX and penetrating tumor cells	[227]
	PTX	The His-AAP-PTX conjugate inhibited the proliferation of tumor cells in an animal model	[228]
	Cisplatin and FA	Cisplatin conjugated with FA and AAP presented an increased antitumor efficiency	[229,230,231]
**SF**	DOX and FA	A nanogel based on SF and FA was used for directing DOX in tumor tissue	[257,270]
	5-FU, cisplatin, DOX, DTX, floxuridine, GEM, MTX, PTX or tamoxifen	The potential antitumor activity of different molecules (5-FU, cisplatin, DOX, DTX, floxuridine, GEM, MTX, PTX or tamoxifen) loaded on SF ± SELP NPs was investigated on cell lines or in animal models	[251,271,272,273,274]
	Curcumin, resveratrol, triptolide/celastrol, emodin, α-mangosteen	Some bioactive compounds incorporated in SF were tested: curcumin (liver, colorectal and breast cancer), resveratrol (colon cancer), triptolide/celastrol (pancreatic cancer), emodin (breast cancer), α-mangosteen (colon and breast cancer)	[251,274]

## Data Availability

Not applicable.

## Abbreviations

3D	three-dimensional
5-FU	5-fluorouracil
AAP	*Auricularia auricula* polysaccharides
ADC	antibody-drug conjugate
ADEPT	antibody-directed prodrug therapy
ADH	adipohydrazide
CDDP	*cis*-diamminedichloroplatinum (II)
CTR	centyrin
CTS	chitosan
CTS-NPs	Hase-chitosan NPs
CHS	cholesterol
CM-PL	carboxymethyl pullulan
DA	deoxycholic acid
DEX	Dextran
DOX	doxorubicin
DS	dextran-spermine
DSPIONs	dextran-coated superparamagnetic iron oxide NPs
DTX	docetaxel
EPR	enhanced permeability and retention
FA	folic acid
FGFR1	fibroblast growth factor receptors
FR	folate receptor
GDEPT	gene-directed enzyme prodrug
GEM	gemcitabine
Gly	glycine
HA	hyaluronic acid
Hase	hyaluronidase
HEP	heparinHis histidine
HPMA	2-hydroxypropyl methacrylate
IC50	half-maximal inhibitory concentration
Ile	isoleucine
KIT	tyrosine-protein kinase
Leu	leucine
LPNPs	lipid-polymer hybrid nanoparticles
LYVE-1	lymphatic vessel endothelium receptor-1
MMP-2	matrix-metalloproteinase-2
MMP-9	matrix-metalloproteinase-9
MTX	methotrexate
MYD88	myeloid differentiation factor 88
NP(s)	nanoparticle(s)
oDex	oxidized dextran
PDC	polymer–drug conjugates
PDEPT	polymer-directed enzyme prodrug therapy
PDGFR1	platelet-derived growth factor receptors
PDTC	pyrrolidine dithiocarbamate
PeDC	Peptide–drug conjugate
PELT	polymer enzyme liposome therapy
PFEP	poly{[9,9-bis(6′-(*N*,*N*,*N*-diethylmethylammonium)hexyl) 2,7-fluorenylene ethynylene]-alt-co-[2,5-bis(3′-(*N*,*N*,*N*-diethylmethylammonium)-1′-oxapropyl)-1,4-phenylene]} tetraiodide
Phe	phenylalanine
PL	pullulan
PLA	pullulan acetate
PrDC	protein-drug conjugate
Pro	proline
PTX	paclitaxel
RHAMM	receptor for hyaluronan-mediated motility
S	sorafenib
SELP	silk-elastin-like polymer
SF	silk fibroin
TiO_2_ NPs	titanium dioxide nanoparticles
TLR4	toll-like receptor 4)
Val	valine
VDEPT	virus-directed enzyme prodrug therapy
VEGFR	vascular endothelial growth factor
